# Effects of Standardised Fermented Papaya Gel on Clinical Symptoms, Inflammatory Cytokines, and Nitric Oxide Metabolites in Patients with Chronic Periodontitis: An Open Randomised Clinical Study

**DOI:** 10.1155/2016/9379840

**Published:** 2016-02-10

**Authors:** Zaira F. Kharaeva, Lyana R. Zhanimova, Magomet Sh. Mustafaev, Chiara De Luca, Wolfgang Mayer, Jeffrey Chung Sheun Thai, Rebecca Tiew Siok Tuan, Liudmila G. Korkina

**Affiliations:** ^1^Kabardino-Balkar Berbekov's State University, 176 Chernishevskogo Street, Nal'chik 360000, Russia; ^2^Medena AG, 16 Industriestrasse, 8910 Affoltern am Albis, Switzerland; ^3^Natural Health Farm, 39 Jalan Pengacara U1/48, Temasya Industrial Park, 40150 Shah Alam, Selangor, Malaysia; ^4^Centre of Innovative Biotechnological Investigations “Nanolab”, 197 Vernadskiy Prospekt, Moscow 119571, Russia

## Abstract

The clinical efficacy of topical administration of standardised fermented papaya gel (SFPG), known to have antioxidant and anti-inflammatory properties,* versus* conventional therapy was evaluated in a group of 84 patients with moderate-to-severe periodontitis, randomly assigned to control group (*n* = 45) undergoing traditional pharmacologic/surgical protocols or to experimental group (*n* = 39), additionally treated with intragingival pocket SFPG (7 g) applications (15 min daily for 10 days). Patients undergoing SFPG treatment showed significant (*P* < 0.05), durable improvement of three major clinical indices of disease severity: reduced bleeding (day 7), plaque and gingival conditions (day 14), and consistent gingival pocket depth reduction (day 45). Proinflammatory nitric oxide metabolites reached normal values in plasma (day 14) and gingival crevicular fluid (GCF) at day 45 with SFPG applications compared to controls that did not reach normalisation. Levels of highly increased proinflammatory (IL-1B, IL-6) and suppressed anti-inflammatory (IL-10) cytokines normalised in the SFPG group by days 14 (plasma) and 45 (GCF), but never in the control group. Although not acting directly as antibiotic, SFPG acted in synergy with human granulocytes blocking adaptive catalase induction in* S. aureus* in response to granulocyte-derived oxidative stress, thus enhancing intracellular bacterial killing.

## 1. Introduction

Epidemiological studies have revealed that more than two-thirds of the world population suffer from one of the chronic forms of periodontal disease [1]. Periodontitis is a chronic inflammatory infectious pathology caused by dental plaque bacteria. The infection-induced inflammatory process leads to progressive destruction of the tissues supporting the teeth, such as gum, periodontal ligament, cementum, and alveolar bone. Periodontitis is currently regarded as a dysbiotic inflammatory disease with a negative impact on both oral and extraoral sites [2, 3]. Thus, the association of chronic periodontitis with several severe pathologies characterised by chronic inflammation, such as rheumatoid arthritis [4–6], diabetes [7], metabolic syndrome [8], and psoriasis [9], has been demonstrated and widely discussed in order to understand whether periodontitis is a causal factor for the initiation and maintenance of these diseases or a consequence of long-lasting generalised inflammatory* status*.

The chronic inflammation of periodontal disease is induced by polymicrobial infection [10, 11], with a leading pathogen being* Porphyromonas gingivalis* [12]. The several hundred of microbes hosted in the oral cavity are commonly organised in “complexes,” that is, associations typically forming biofilms in gingival pockets and supragingival plaque [13], which makes microbes more pathogenic and less sensitive to traditional antibiotic therapies [14]. The microbes become resistant to antibiotics owing to their innate or acquired capacity of adaptation to both antimicrobial drugs and host defence [15–18]. For example, to overcome oxidative stress with bactericidal action, the induction of microbial antioxidant systems, mainly, through OxyR and Sox(R + S) regulatory mechanisms occurs [15, 19–21].

The presence of distinct microbes in the periodontal environment has been associated to increased levels of host-produced proinflammatory cytokines, such as tumour necrosis factor-*α* (TNF-*α*) and interleukin-6 (IL-6), in the gingival fluid and tissue, that define the severity of destructive processes in both the gingival epithelium and bone tissue [11]. Interleukin-1 beta, a potent stimulator of bone resorption, was hypothesised to be implicated in the pathogenesis of periodontal tissue destruction [22]. It has been suggested that the local gingival cytokines [23] and/or low-level asymptomatic bacteria and endotoxemia [24] may affect the plasma concentration of proinflammatory biomarkers such as systemic cytokines, adipokines, high-sensitivity C-reactive protein, products of lipid peroxidation, and reactive oxygen and nitrogen species (ROS and RNS, resp.) [25–28]. Periodontitis severity and bone resorption markers, such as osteoprotegerin levels and receptor activator of nuclear factor kappaB (NF*κ*B) ligand, were closely associated with total oxidant balance in serum and gingival crevicular fluid (GCF) [28].

Although many efforts have been made to develop clinically effective and cost-effective therapies, none has proven to have sufficient efficacy, and the urgent need for better treatments and preventive strategies remains.

In the folk medicine of South East Asia, papaya preparations have been traditionally used as antimicrobial, anti-inflammatory, and wound healing remedies. Standardised fermented papaya preparations have shown remarkable free radical scavenging, antioxidant, anti-inflammatory, phagocyte-stimulating, and wound healing activities in the* in vitro* and* in vivo* experiments [29, 30], as well as in clinical trials [31]. Of particular interest, oral supplementation with fermented papaya preparations significantly corrected metabolic and oxidative stress markers in patients with type 2 diabetes [32, 33]. In the present clinical laboratory study, a standardised fermented papaya gel (SFPG, Carica Co., Manila, The Philippines) was used for topical applications to patients with moderate-to-severe periodontitis. Effects on local (GCF) and systemic (plasma) levels of inflammatory cytokines and nitric oxide metabolites were compared with clinical improvements. A novel synergic mechanism of antibacterial action of human leukocytes and the papaya gel was found.

## 2. Materials and Methods

### 2.1. Patients and Study Design

The study enrolled a group of 84 patients of both sexes (age range 38–62 years) suffering from chronic moderate-to-severe periodontitis and visiting dentists at Medical Department of the Kabardino-Balkar Berbekov's State University (Nal'chik, Russian Federation). The study protocol was scrutinised and approved by the local Ethical Committee (Protocol MD-023-2013). The patients were randomly assigned to experimental or control groups. The demographic distribution of periodontitis patients in the groups is shown in Table 1. All of them were treated by traditional therapeutic and surgical protocols, if needed.

Traditional treatment protocols included education to oral hygiene, plaque removal, teeth enamel polishing, and surgical elimination of tartar. In addition, local instillations of 0.06% chlorohexidine gluconate into periodontal pockets were regularly performed as antimicrobial and anti-inflammatory procedures.

The patients of the experimental group additionally received intragingival pocket applications (7 g of SFPG per patient per day) for 10 consecutive days. The SFPG was kept inside the pocket for 15 min and then washed out by physiological solution. Since SFPG consists of a complex mixture of fermented papaya fruit ingredients each of which possesses a biological activity, and water as a solvent of these organic active agents of plant origin, we omitted the use of pure water as a placebo in the controls group.

Healthy donors matched by sex and age (*n* = 25) were recruited from the Medical Department staff and trainees, who donated blood and GCF after signing the informed consent form.

Subjects with severe chronic and/or infection diseases in acute phase, as well as virus hepatitis patients, were excluded from the study. No patients and controls entering the study had taken any drugs or nutraceutical supplements known to interfere with the redox* status* or inflammation since at least six weeks. No alcohol- or drug-abusers or smokers were present in any of the three cohorts studied. All subjects consented to personal and anamnestic data collection and biological material sampling.

### 2.2. Clinical Assessment

Clinical efficacy of intragingival applications of SFPG was assessed by subjective evaluation of doctors and patients and objective clinical indices of chronic periodontitis severity [34, 35]. These latter included the gingival and plaques indexes, in accord with Löe and Silness method, the percentage of sites bleeding on probing by Muhlemann's index, and the state of gingival inflammation by Parma's papillae-gum margin-alveolar (PMA) index. The gingival pocket depth was measured by periodontal probing with a sensor connected to the automatic computerised diagnostic system Florida Probe (Florida Probe Co., FL, USA). All the indexes were repeatedly determined at days 0, 7, 14, and 45 of the clinical study. The level of destruction of periodontal bone structures was recorded twice, in the beginning and at day 45 of the trial, using orthopanoramic images (Orthophos XG5, Sirona Ltd., Germany) and computer tomography on a High Speed DX-i (General Electric Healthcare, WI, USA).

### 2.3. Biological Material Collection and Processing

A Whatman number 1 sterile paper bar of 3 mm width was carefully introduced into the tooth pocket and kept in place for 2 min. Then, the filter paper bar was transferred into vials containing 2 mL phosphate buffer solution. The procedure was carried out for all teeth affected by periodontitis, and the content of vials was finally pooled. The collected samples of GCF fluid were stored at −80°C until being analysed for nitrite/nitrate and cytokine contents.

Peripheral venous blood (20 mL) was drawn after overnight fasting into vacutainers with ethylene diamine tetraacetic disodium salt (EDTA) as anticoagulant. Both patient and donor samples were processed and analysed in parallel. Circulating polymorphonuclear leukocytes (PNM) were obtained by double density gradient centrifugation of 15 mL of total blood (Histopaque, *d* = 1.077 and 1.199 g/mL). PMN from the interface were resuspended in phosphate buffer saline, centrifuged at 1650 rpm for 10 min, and then aliquoted at 5 × 10^6^/vial. Freshly isolated PMN were used in phagocytosis assays and measurements of reactive oxygen species production. Samples of whole blood (5 mL) were allowed to sediment for 40 min at room temperature and then plasma supernatant was collected, aliquoted, and stored at −80°C until quantitative analyses of nitrites/nitrates and cytokines.

### 2.4. Reagents and Assay Kits

The majority of chemical reagents and solvents, H_2_O_2_ standard, mediums for human and bacterial cells cultivation, and the chemiluminescence probe luminol were purchased from Sigma Chemical Co. (St. Louis, MO, USA); kits for enzyme activities and nitrite/nitrate assays were from Cayman Chem. Co. (Ann Arbor, MI, USA); monoclonal antibodies for ELISA interleukin kits were from R&D Systems (Minneapolis, MN, USA); kits for protein determination were from Bio-Rad Laboratories (Bio-Rad Inc., Hercules, CA, USA).

### 2.5. Bacterial Strains, Growth Conditions, and Exposure to SFPG

Bacterial strains used in this study were as follows:* Staphylococcus aureus* strains 241 and 242 (high catalase-containing strains), 548 (low catalase-containing strain), and 556 (no-catalase strain).* S. aureus* were grown in tryptic soy broth at 37°C under continuous shaking as described previously [19]. To assess effects of SFPG on the enzymatic activity in bacteria, and on their phagocytosis by PMN, a suspension of bacteria (1 mL, 10^9^ cells/mL) was mixed with the same volume of 0.9% SFPG and coincubated for 1 hour at 37°C under shaking conditions at 180 rpm. Finally,* S. aureus* cultures were thoroughly washed trice by big volumes of fresh physiological solution.

### 2.6. Phagocytosis and Postphagocytosis Bacterial Survival Assays

Intensity of phagocytosis was assessed by routine clinical bacteriology methods. Briefly, 1 mL of PMN suspension (10^6^ cells/mL) was mixed with 1 mL of bacterial suspension (10^7^ cells/mL). The mixture was incubated under continuous shaking at 37°C for 30 min. Then, smears were prepared on microscopic slides, fixed, and stained by Romanowsky-Giemsa dye. The smears were examined under microscope, and percent of phagocytozing PMN was determined. The remaining mixture was used to assess intracellular bacterial killing. After centrifugation at 1500 ×g for 10 min, bacterial sediments were collected and diluted to an OD_600_ of 0.1 with fresh medium, spread onto Petri dishes with appropriate medium with agar, and were allowed to grow at 37°C for 24 hours. Bacteria survival rates were calculated as colony-forming-units (CFU) of cells coincubated with granulocytes, divided for that of untreated bacteria. The results were expressed in percent.

### 2.7. Bacterial Catalase Assay

Bacterial cells pretreated and nonpretreated with SFPG were grown in appropriate mediums to an OD_600_ of 0.3, collected, centrifuged, and lysed with a Pierce bacterial extraction reagent. Cell debris was removed by centrifugation and catalase activity in the supernatant was measured by Aebi's method [36], using a freshly prepared H_2_O_2_ for a standard curve. Protein content was measured according to Bradford, using the Bio-Rad microplate assay kit (Bio-Rad Laboratories, Inc., Hercules, CA, USA). One unit of catalase activity was defined as the decomposition of 1 *μ*mol H_2_O_2_ per min at pH 7.0 at 25°C. The results were expressed in Units/g protein.

### 2.8. Redox Assays

Whole blood and PMN luminol-dependent chemiluminescence (CL, expressed in counts per second/*μ*L or cps/10^6^ cells, resp.) response to different strains of* S. aureus* was quantified by a Victor^2^ 1420 counter, equipped with Wallac 1420 Software (Perkin Elmer, MA, USA). In brief, 10^5^ cells or 10 *μ*L of whole blood was added to 1 mL of preheated Hank's Balanced Salt Solution (pH 7.4) containing 0.2 mM luminol. Then, 10^7^/mL of* S. aureus* was added and the chemiluminescence response was monitored for 5 min. Each measurement was repeated three–five times. Results were expressed in cps per 1 mL of whole blood or 10^6^ PMN.

Superoxide radical production by PMN was measured by the superoxide dismutase- (SOD-) dependent cytochrome c reduction method described previously [37] and expressed as *μ*mol/min/10^6^ PMN.

Plasma and GCF levels of nitrites/nitrates (NO_2_^−^/NO_3_^−^, expressed as *μ*moles/L) were measured spectrophotometrically by Griess reagent kit, following manufacturer's instructions.

### 2.9. Cytokine Assays

The plasma and GCF levels of interleukin-1*β* (IL-1*β*,) IL-6, and interleukin-10 (IL-10) were measured by enzyme-linked immunosorbent assay (ELISA) purchased from R&D Systems (Minneapolis, MN, USA), following manufacturer's instructions. Cytokine concentrations were expressed in pg/mL of plasma, and each protein factor was quantified in the linear range of its calibration curve.

### 2.10. Statistical Analysis

All biochemical measurements were done in triplicate and data were statistically evaluated. Statistical analysis of clinical and laboratory data was performed using STATISTICA 6.0 program (StatSoft Inc., Tulsa, OK, USA). Reported data were treated as continuous. Normality of data was checked using the Shapiro-Wilk test. Since the distribution of the data in the three groups was significantly different from normal, nonparametric statistics was used. Values were presented as mean ± standard error of the mean of triplicate analyses. The Mann-Whitney *U* test for independent samples was employed for comparison between periodontitis groups and healthy controls. Significance was assumed at a *P* value < 0.05.

## 3. Results

### 3.1. Clinical Efficacy of Standardised Fermented Papaya Gel Application

**Figure 1:**
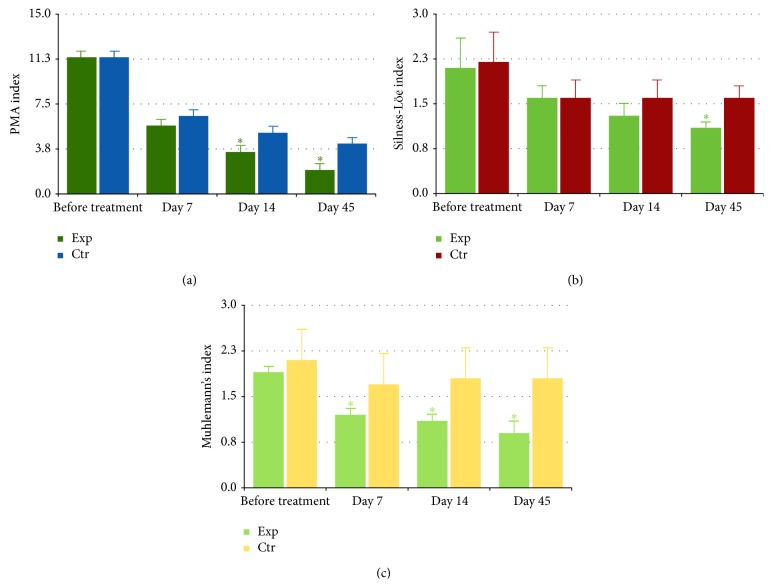
Dynamics of the clinical indexes of periodontitis severity: PMA (clinical attachment and pocket depth index) index (a); Silness-Löe (gingival and plaque) index (b); Muhlemann's (bleeding on probing) index (c). The experimental group (Exp, *n* = 39) was treated with conventional periodontal treatment plus topical daily application of fermented papaya gel into gingival pockets; the control group (Ctr, *n* = 45) received conventional periodontal treatment for the same period of time. Values are represented as mean and standard deviation of the mean (SD). ^*∗*^ *P* < 0.05* versus* Ctr.

The topical administration of the standardised fermented papaya gel (SFPG) proved to be nontoxic and well tolerated by the patients, among which no adverse effect or withdrawal from trial was registered throughout the whole study. Recruited patients assigned to both control and experimental groups exhibited clinical features of moderate-to-severe periodontitis that was revealed by visual clinical observation and by assessment with conventional objective indexes of gingival and periodontal conditions, that is, PMA, Silness-Löe, and Muhlemann's indexes (Figure 1). The baseline values of the three clinical indexes did not differ in the experimental and control groups. The indexes of the periodontitis severity gradually subsided upon administration of conventional nonsurgical methods of treatment. Statistically significant (*P* < 0.05) difference of Muhlemann's index (gingival bleeding on probe) between the group treated additionally with SFPG and the control one appeared by the 7th day, was observed for the entire period of SFPG administration (10 days), and persisted in the follow-up period at the 14th and 45th days (Figure 1(c)). Similar differences for PMA index (inflammatory marker) appeared by day 14 (Figure 1(a)), while for Silness-Löe index (plaque* status* and gingival pocket depth) they were found later, by day 45 during the follow-up period (Figure 1(b)).

Decreased levels of periodontal bone resorption were evident for the experimental group patients by day 45, when second orthopanoramic images were registered, while no differences in periodontal bone conditions were observed in the control group of patients during the study course (data not shown).

### 3.2. Effects of Standardised Fermented Papaya Gel Applications on Nitrite/Nitrate Content in Gingival Crevicular Fluid (GCF) and Blood Plasma

**Figure 2:**
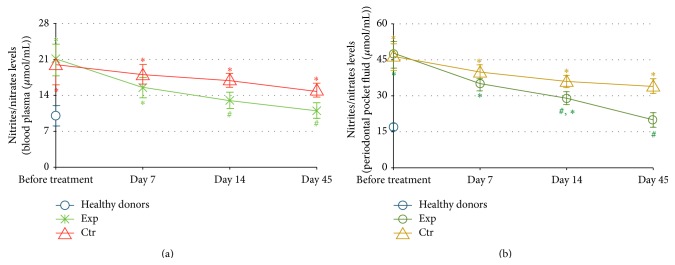
Dynamics of nitrite and nitrate levels (*μ*mol/mL) in plasma (a) and crevicular periodontal fluid (b). The experimental group (Exp, *n* = 39) was treated with conventional periodontal treatment plus topical daily application of fermented papaya gel into gingival pockets; the control group (Ctr, *n* = 45) received conventional periodontal treatment for the same period of time. Healthy donors: *n* = 25. Values are represented as mean and standard deviation (SD). ^#^ *P* < 0.05* versus* analogous day of treatment in the control group; ^*∗*^ *P* < 0.05* versus* healthy donors.

The dynamics of nitrite/nitrate levels in GCF and blood serum is shown in Figure 2. The data of periodontitis patients were compared with those of healthy donors (*n* = 25) matching in age and sex. The concentration of NO_2_^−^/NO_3_^−^ in GCF was highly elevated in periodontitis* versus* normal values (Figure 2(b)). Also, plasma levels of NO_2_^−^/NO_3_^−^ were statistically greater in the patients than in healthy subjects (Figure 2(a)). At the same time, baseline concentrations of NO_2_^−^/NO_3_^−^ did not differ for the experimental and the control groups. The levels of this proinflammatory marker gradually diminished in the course of therapy and the follow-up period for both periodontitis groups. However, the local and systemic NO_2_^−^/NO_3_^−^ levels did not reach normal values in the control group over the entire period of observation, while plasma and GCF concentrations of NO_2_^−^/NO_3_^−^ were normalised in the experimental group by the 14th and 45th days, respectively. Of note, the difference (*P* < 0.05) between the GCF levels of NO_2_^−^/NO_3_^−^ for two groups reached statistical significance by the 14th day of the trial.

### 3.3. Effects of Standardised Fermented Papaya Gel Applications on Pro- and Anti-Inflammatory Cytokines in Gingival Crevicular Fluid (GCF) and Blood Plasma

**Figure 3:**
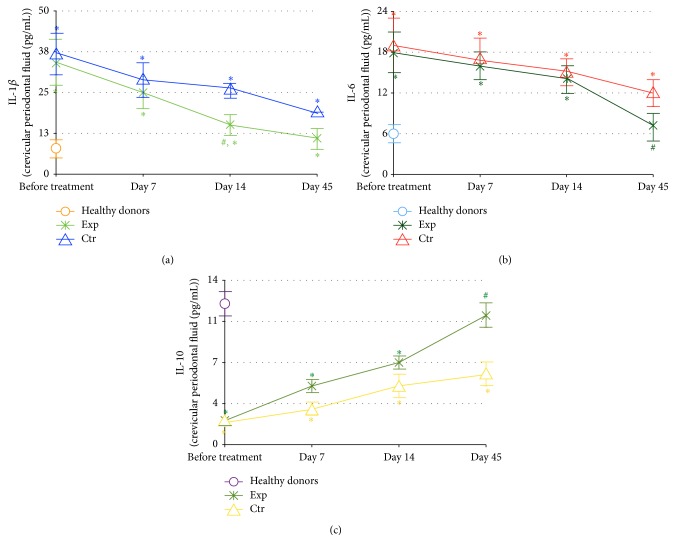
Dynamics of the pro- and anti-inflammatory cytokines IL-1*β* (a), IL-6 (b), and IL-10 (c) in the crevicular periodontal fluid (pg/mL). The experimental group (Exp, *n* = 39) was treated with conventional periodontal treatment plus topical daily application of fermented papaya gel into gingival pockets; the control group (Ctr, *n* = 45) received conventional periodontal treatment for the same period of time. Healthy donors: *n* = 25. The content of cytokines in the crevicular periodontal fluid was quantified by ELISA method using corresponding monoclonal antibodies. Values are represented as mean and standard deviation of the mean (SD). IL-1*β*: interleukin 1*β*; IL-6: interleukin 6; and IL-10: interleukin 10. ^*∗*^ *P* < 0.05* versus* analogous day of treatment in the control group. ^#^ *P* < 0.05* versus* healthy donors.

**Figure 4:**
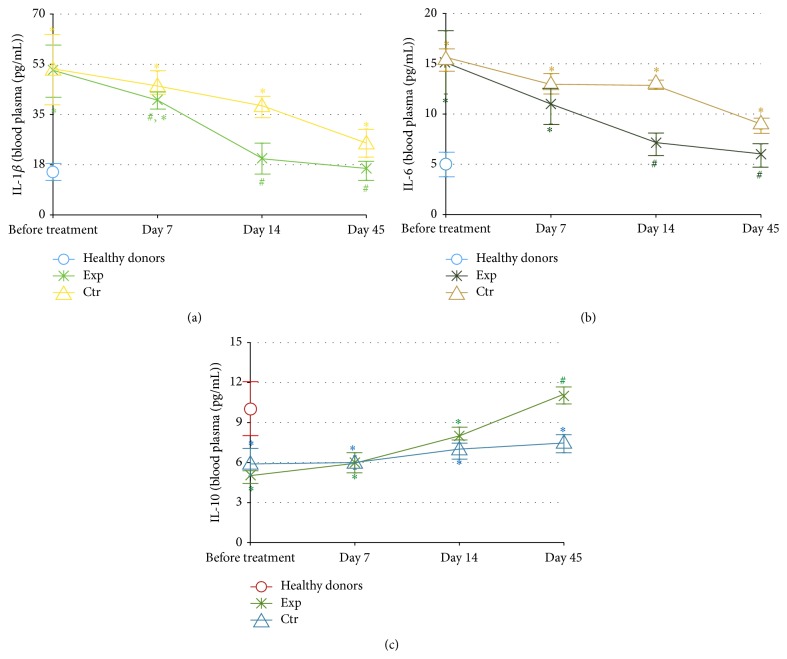
Dynamics of the pro- and anti-inflammatory cytokines IL-1*β* (a), IL-6 (b), and IL-10 (c) in the plasma (pg/mL). The experimental group (Exp, *n* = 39) was treated with conventional periodontal treatment plus topical daily application of fermented papaya gel into gingival pockets; the control group (Ctr, *n* = 45) received conventional periodontal treatment for the same period of time. Healthy donors: *n* = 25. The content of cytokines in plasma was quantified by ELISA method using corresponding monoclonal antibodies. Values are represented as mean and standard deviation of the mean (SD). IL-1*β*: interleukin 1*β*; IL-6: interleukin 6; and IL-10: interleukin 10. ^#^ *P* < 0.05* versus* analogous day of treatment in the control group. ^*∗*^ *P* < 0.05* versus* healthy donors.

Quantification and dynamics of GCF and plasma levels of two proinflammatory cytokines (IL-1*β* and IL-6) and of the anti-inflammatory cytokine IL-10 are shown in Figures 3 and 4, respectively, and compared with the corresponding cytokine levels in healthy subjects. In the GCF (Figure 3), the background levels of both IL-1*β* and IL-6 were highly and equally elevated (Figures 3(a), and 3(b)), while IL-10 values were decreased in the periodontitis groups as compared to healthy controls (Figure 3(c)). In the course of the clinical study, the GCF cytokine concentrations tended to normalisation reaching normal levels by day 45 in the patients of experimental group treated with SFPG. On the other hand, normal cytokine levels in GCF were never achieved in the control group, and statistical intergroup difference of IL*β* content was observed by day 14.

Similar results were obtained for the dynamics of plasma levels of the same cytokines (Figure 4). However, the normality for IL-1*β* and IL-6 levels was reached earlier by the 14th day, and the difference between the groups became evident by day 7 (IL-*β*, Figure 4(a)), by day 14 (IL-6, Figure 4(b)), and by day 45 (IL-10, Figure 4(c)).

### 3.4. Effects of Standardised Fermented Papaya Gel on* S. aureus* Resistance towards Oxidative Stress Induced by Human Granulocytes

To prove the hypothesis that the comparatively high and durable clinical efficacy and the positive biochemical and molecular anti-inflammatory effects of SFPG towards chronic periodontitis might be partly explained by its antibacterial properties, the experimental part of the research was designed using* in vitro* systems containing isolated human granulocytes and* S. aureus* strains.

The SFPG did not exhibit direct bacteriostatic or bactericidal action towards* S. aureus* since its incubation with the bacteria did not affect number of colony-forming-units (data not shown). When human granulocytes were mixed with several strains of* S. aureus* (the ratio of human cells to microbes was 1 : 100) which differed by catalase expression, all these strains even without opsonisation induced ROS production by phagocytes, assessed both by luminol-dependent chemiluminescence (a semiquantitative assay for total H_2_O_2_ decomposition products, such as hydroxyl radicals, hypochlorite, and peroxynitrite) and by the spectrophotometric superoxide assay (Table 2). While the intensity of luminol-dependent chemiluminescence inversely correlated with catalase activity in the strain (correlation coefficient, *r* = −0.82, *P* < 0.05), superoxide release from phagocytes was constant and not connected with catalase activity. Of particular importance, although catalase activity in* S. aureus* did not influence the intensity of phagocytosis (data not shown), it strongly and directly correlated with bacterial survival after phagocytosis (correlation coefficient *r* = 0.9, *P* < 0.05).

In the following set of experiments, two strains of* S. aureus* with elevated or absent catalase activities were pretreated with SFPG for 1 hour and then mixed with human granulocytes (Table 3). Initially high activity of the catalase enzyme, decomposing hydrogen peroxide to molecular oxygen and water, was significantly suppressed and luminol-dependent chemiluminescence and intracellular killing of* S. aureus* were remarkably enhanced, although the intensity of phagocytosis did not change. As expected, SFPG pretreatment of bacterial strain lacking catalase did not induce any significant change in the intracellular killing or ROS production.

## 4. Discussion

Periodontitis, being one of the most frequent chronic inflammatory pathologies with as yet unmet therapeutic needs, has been attracting increasing attention recently, due to its evident impact on the development of systemic low-level inflammation leading to metabolic syndrome, a comorbidity characteristic for cardiovascular diseases, diabetes, obesity, arthritis, and so forth [38, 39]. Molecular pathways involved in the pathogenesis of persistent oral inflammation and rapid destruction of epithelial lining, soft and bone tissues, are far from being fully understood. In this clinical laboratory investigation, we attempted to reveal peculiarities of local and systemic chronic inflammation in periodontitis and to evaluate a remedy to attenuate clinical symptoms and molecular features of the inflammatory process as well as to diminish bacterial overload in the periodontal area. A commercially available SPFG with reported antibacterial, antioxidant, anti-inflammatory, and antimetabolic syndrome properties [40–44] was evaluated clinically. Topical administration of the gel was nontoxic and well tolerated by the participants and resulted in satisfactory and durable clinical efficacy assessed by objective measurements of periodontitis severity indices (Figure 1).

Local and generalised oxidative stress since long time have been considered as molecular hallmarks of the disease. An increased lipid peroxidation and decreased antioxidant activity have been found in saliva, GCF, and blood plasma of patients with periodontitis [45, 46]. Later, the correlation between the disease severity and increased lipid peroxidation assessed by malonyl dialdehyde (MDA) content has been reported [47]. Based on the data that plasma MDA content was superior compared to saliva, the authors have concluded that periodontitis was associated with systemic oxidative stress. In a recent comparative clinical study, the highest levels of serum MDA and 8-hydroxy-2′-deoxyguanosine (8-OHdG) have been recorded in patients with a combination of periodontitis and hyperlipidemia [8]. Our findings that background levels of nitrites and nitrates, reflecting nitric oxide overproduction and an elevated risk of peroxynitrite formation, were greatly increased compared to normal values both locally in the gingival crevicular fluid and systemically in all patients with moderate-to-severe form of periodontitis (Figure 2) well correlate with the previous publications on oxidative stress as a characteristic feature of periodontitis and metabolic syndrome [48]. The risk of nitrosative stress and of its inflammation-promoting effects was attenuated by SFPG application as compared to conventional protocols. This antioxidant action was durable as it lasted for at least 35 days after cessation of the SFPG applications. Chronic local and systemic inflammation in periodontitis was confirmed by the remarkably increased initial levels of major proinflammatory cytokines (IL-1*β* and IL-6) and suppressed level of anti-inflammatory cytokine IL-10 involved in the development of periodontitis and its severity in both GCF and blood plasma (Figures 3 and 4). Again, the therapeutic course of SFPG led to complete and durable normalisation of cytokine* status* both locally and systemically. Previous publications have shown that the marked reduction of clinical parameters corresponded to decreased levels of IL-1*β* and thiobarbituric acid reactive substances in the crevicular fluid after successful periodontal treatments [22, 39]. Levels of IL-1*β* and IL-10 in GCF of patients with chronic periodontitis have been significantly altered in diseased* versus* nondiseased sites and tended to return to normal values after a 32-week periodontal therapy. Higher-than-normal amount of IL-1*β* before therapy correlated with disease severity, while an inverse relationship between IL-1*β* and IL-10 was evident [49]. Similar conclusions regarding salivary IL-1*β* dynamics have been drawn in a case-control clinical study enrolling the patients before and after successful periodontal therapy [50]. These findings allowed suggesting a close association between salivary IL-1*β* and severity of disease.

Successful treatments of periodontal disease and reduction of oral inflammation have shown positive effects on distinct markers of metabolic syndrome which mainly reflect chronic systemic inflammation in type 2 diabetes [27]. Our data seem to confirm previous findings that orally administered fermented papaya products were especially effective against inflammatory and metabolic markers in type 2 diabetes in humans [32] and animal models of diabetes [51]. The mode of topical oral application of SFPG we used in the present study showed more pronounced local anti-inflammatory and antimetabolic effects than the systemic ones (compare Figure 2(a) with Figure 2(b) and Figure 3 with Figure 4). Recent publication [52] has reported that saliva and its major components, such as albumin and mucin, substantially increase antioxidant capacity of another fermented papaya preparation by solubilisation of plant-derived polyphenols. It could be also assumed that a combination of intragingival with oral dietetic administration of SFPG to patients with periodontitis could better alleviate generalised chronic inflammation and connected metabolic disorders caused by the oral pathology.

Since polymicrobial infection is generally accepted as a major etiological factor in periodontitis, the primary lines of treatment include mechanical removal of microbial biofilms as well as systemic and topical antibiotics [52]. The need for repeated painful cleaning procedures and the acquired resistance of dental bacteria to antibiotics prompted an extensive search for alternative, nontoxic, clinically effective, and cost-effective remedies to decrease local bacterial overload. Recently, molecules inhibiting biofilm formation have been suggested as having therapeutic potential in the treatment of chronic infectious diseases like periodontitis resistant to conventional antibiotics. Among the most effective disrupters of microbial biofilms, flavonoids, 2-aminoimidazole alkaloids, and halogenated furanones of plant origin have been identified [53].

**Figure 5:**
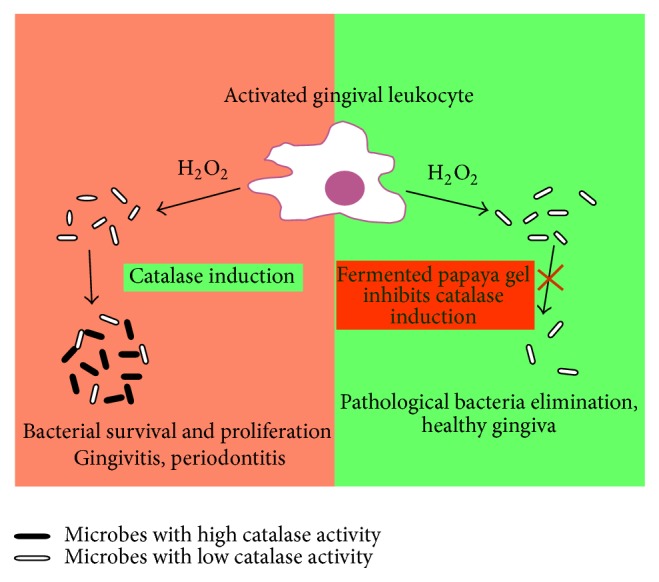
Standardised fermented papaya gel (SFPG) inhibits bacterial survival by the suppression of their adaptive antioxidant defence. Catalase constitutively expressed in pathogenic bacteria including* Porphyromonas gingivalis* could be further induced after phagocytosis due to intracellular oxidative stress in granulocytes/tissues macrophages. The adaptive catalase induction through bacterial redox sensitive regulons leads to reduced bacterial killing by reactive oxygen species and to their survival and dissemination (infection persistence). Thus, chronic bacteria-driven inflammatory process persists in the periodontal tissues (gingivitis, periodontitis). SFPG blocks, by unknown as yet mechanism, the adaptive antioxidant response of pathogenic bacteria to oxidative burst of granulocytes/tissue macrophages. Hence, bacteria become more sensitive to oxidative killing. Thus, bacterial load diminishes and the inflammatory process in the periodontal tissues subsides.

The generation of ROS (superoxide radicals and H_2_O_2_) by granulocytes is a key host defence against* Staphylococcus aureus*,* Escherichia coli*,* Porphyromonas gingivalis*, and other pathogenic bacteria [18–20, 54]. Enhanced bacterial resistance to granulocyte-produced ROS partly depends on the induction of antioxidant enzymes including catalase and superoxide dismutases [20, 54–56]. Molecular mechanisms of the adaptive response to oxidative stress in bacteria are complex and similar to those in mammalian cells. They are controlled by key transcriptional regulators, such as OxyR (analogous to Nrf2 in mammalians) and PerR sensing hydrogen peroxide when it oxidises thiolates or iron moieties, respectively [19–21, 57], while SoxR and SoxS seem to control levels of redox-cycling compounds [57]. OxyR represents the redox-regulatory protein identified in most Gram-negative and some Gram-positive bacteria and other bacterial species, including* P. gingivalis*. OxyR is required to induce oxidative stress-related genes, such as* sod* (encoding superoxide dismutases),* kat* (encoding catalase),* pdgX* (encoding peroxiredoxin-glutaredoxin), and* ahpC* (encoding alkyl hydroperoxide reductase) [19–21, 58, 59]. Recently, additional OxyR-independent mechanism of oxidative stress resistance and virulence in* P. gingivalis* connected to a transcriptional regulation by PG2212 zinc finger protein has been described [60]. The antioxidant enzymes are localised periplasmically in a wide range of bacteria, could be released from them, and appear to protect bacterial survival from intracellularly and extracellularly produced ROS [58]. The hypothesis that antibiotics generate lethal oxidative stress in microbes has become an attractive mechanistic explanation of their clinical efficacy. However, a strong argument has also been raised that sufficient clinical efficacy of such antibiotics cannot be achieved without simultaneous block of inducible antioxidant defence pathways in microbes [61]. SPGF did not possess direct bactericidal activity (data not shown); however, it substantially diminished microbial capacity to adapt to prooxidants, first of all H_2_O_2_ and its toxic metabolites like hypochlorite (OCl^−^) produced by granulocytes, known as key cellular mediators of inflammation in periodontitis [62]. The catalase-containing bacteria pretreated by SFPG induced greater luminol-dependent chemiluminescence response (a measure of hydrogen peroxide and hypochlorite production by granulocytes) than nonpretreated ones (Table 2). In parallel, SFPG blocked completely bacterial catalase induction in* S. aureus*, which correlated with lower bacterial survival after phagocytosis. On the basis of these results, we assume that SPFG could be regarded as exerting an indirect antibiotic action, and a possible adjuvant function with direct antibiotics, owing to its capacity to inhibit bacterial adaptive antioxidant response, such as catalase induction, to granulocyte-induced or antibiotic-induced oxidative stress (Figure 5).

## 5. Conclusions

The present clinical laboratory study showed the remarkable and durable clinical efficacy of topical administration of standardised fermented papaya gel to patients suffering from moderate-to-severe chronic periodontitis. Positive clinical results were associated with a normalisation of the inflammatory/anti-inflammatory cytokine pattern and of the proinflammatory metabolites of nitric oxide in gingival crevicular fluid and blood plasma. Clinical and biochemical anti-inflammatory effects of fermented papaya gel preparation were partly attributed to its indirect antibacterial impact through the inhibition of adaptive antioxidant response in bacteria to intracellular oxidative stress in phagocytes.

## Figures and Tables

**Table 1:** Demographic distribution of periodontitis patients in the treatment groups.

Group	Number of patients	Age, years	Sex	
			M	F
Experimental (conventional periodontal treatment + fermented papaya)	39	39–62	17	22

Control (conventional periodontal treatment)	45	38–60	22	23

**Table 2:** Effects of *S. aureus* strains with different catalase activity on reactive oxygen species production by human granulocytes.

*S. aureus* strain characteristics		Reactive oxygen species production by human polymorphonuclear leukocytes (PMN)	
Strain number	Catalase activity U/2 × 10^7^ cells	Luminol-dependent chemiluminescence cpm/10^6^ PMN	Superoxide production, *μ*mol/min/10^6^ PMN
241	4.9 ± 0.5	1980 ± 147	0.46 ± 0.01
242	5.1 ± 0.5	2340 ± 125	0.49 ± 0.01
548	0.5 ± 0.1	5220 ± 330	0.44 ± 0.01
556	0.0	10620 ± 410	0.42 ± 0.01

**Table 3:** Effects of *S. aureus* pretreatment (+) with standardised fermented papaya gel on their catalase activity, ROS production, phagocytosis, and intracellular killing by human granulocytes.

Parameter (units)	*S. aureus* strain, pretreatment with SFPG			
	*S. aureus*, strain 242 (catalase +) (−) pretreatment	*S. aureus*, strain 242 (catalase +) (+) pretreatment	*S. aureus*, strain 556 (catalase −) (−) pretreatment	*S. aureus*, strain 556 (catalase −) (+) pretreatment
Catalase activity in *S. aureus* (Units/2 × 10^7^ cells)	5.1 ± 0.1	3.3 ± 0.1^*∗*^	0	0
Phagocytosis intensity (%)	38 ± 5	36 ± 6	36 ± 8	34 ± 6
Intracellular killing of *S. aureus* (%)	60.5 ± 0.2	95.8 ± 0.2^*∗∗*^	95.0 ± 0.2	95.7 ± 0.3
ROS production by PMN activated by *S. aureus* (cpm/10^6^ PMN)	2340 ± 125	3230 ± 200^*∗*^	10620 ± 410	10800 ± 830

^*∗*^ *P* < 0.05  *versus* bacteria without pretreatment.

^*∗∗*^ *P* < 0.001  *versus* bacteria without pretreatment.

## References

[1] Petersen P. E., Ogawa H. (2012). The global burden of periodontal disease: towards integration with chronic disease prevention and control. *Periodontology 2000*.

[2] Hajishengallis G., Chavakis T., Hajishengallis E., Lambris J. D. (2015). Neutrophil homeostasis and inflammation: novel paradigms from studying periodontitis. *Journal of Leukocyte Biology*.

[3] Hajishengallis G. (2014). Periodontitis: from microbial immune subversion to systemic inflammation. *Nature Reviews Immunology*.

[4] de Pablo P., Chapple I. L. C., Buckley C. D., Dietrich T. (2009). Periodontitis in systemic rheumatic diseases. *Nature Reviews Rheumatology*.

[5] Wolff L. F. (2014). Diabetes and periodontal disease. *American Journal of Dentistry*.

[6] Demmer R. T., Molitor J. A., Jacobs D. R., Michalowicz B. S. (2011). Periodontal disease, tooth loss and incident rheumatoid arthritis: results from the First National Health and Nutrition Examination Survey and its epidemiological follow-up study. *Journal of Clinical Periodontology*.

[7] Mealey B. L., Oates T. W. (2006). Diabetes mellitus and periodontal diseases. *Journal of Periodontology*.

[8] Fentoğlu Ö., Kırzıoğlu F. Y., Bulut M. T. (2015). Evaluation of lipid peroxidation and oxidative DNA damage in patients with periodontitis and hyperlipidemia. *Journal of Periodontology*.

[9] Sharma A., Raman A., Pradeep A. R. (2015). Association of chronic periodontitis and psoriasis: periodontal status with severity of psoriasis. *Oral Diseases*.

[10] Darveau R. P. (2010). Periodontitis: a polymicrobial disruption of host homeostasis. *Nature Reviews Microbiology*.

[11] Dosseva-Panova V. T., Popova C. L., Panov V. E. (2014). Subgingival microbial profile and production of proinflammatory cytokines in chronic periodontitis. *Folia Medica*.

[12] Zhu Z., Chen W., Hao L. (2015). Ac45 silencing mediated by AAV-sh-Ac45-RNAi prevents both bone loss and inflammation caused by periodontitis. *Journal of Clinical Periodontology*.

[13] Haffajee A. D., Socransky S. S., Patel M. R., Song X. (2008). Microbial complexes in supragingival plaque. *Oral Microbiology and Immunology*.

[14] Socransky S. S., Haffajee A. D., Cugini M. A., Smith C., Kent R. L. (1998). Microbial complexes in subgingival plaque. *Journal of Clinical Periodontology*.

[15] Giuffrè A., Borisov V. B., Arese M., Sarti P., Forte E. (2014). Cytochrome bd oxidase and bacterial tolerance to oxidative and nitrosative stress. *Biochimica et Biophysica Acta*.

[16] Maisonneuve E., Gerdes K. (2014). Molecular mechanisms underlying bacterial persisters. *Cell*.

[17] Douglas C. W. I., Naylor K., Phansopa C., Frey A. M., Farmilo T., Stafford G. P. (2014). Physiological adaptations of key oral bacteria. *Advances in Microbial Physiology*.

[18] Heindorf M., Kadari M., Heider C., Skiebe E., Wilharm G. (2014). Impact of *Acinetobacter baumannii* superoxide dismutase on motility, virulence, oxidative stress resistance and susceptibility to antibiotics. *PLoS ONE*.

[19] Painter K. L., Strange E., Parkhill J. (2015). *Staphylococcus aureus* adapts to oxidative stress by producing H_2_O_2_-resistant small-colony variants via the SOS response. *Infection and Immunity*.

[20] Xie H., Zheng C. (2012). OxyR activation in *Porphyromonas gingivalis* in response to a hemin-limited environment. *Infection and Immunity*.

[21] Zhang L., Alfano J. R., Becker D. F. (2015). Proline metabolism increases *katG* expression and oxidative stress resistance in *Escherichia coli*. *Journal of Bacteriology*.

[22] Tüter G., Kurtiş B., Serdar M. (2001). Interleukin-1*β* and thiobarbituric acid reactive substance (TBARS) levels after phase I periodontal therapy in patients with chronic periodontitis. *Journal of Periodontology*.

[23] Prabhu A., Michalowicz B. S., Mathur A. (1996). Detection of local and systemic cytokines in adult periodontitis. *Journal of Periodontology*.

[24] Scannapieco F. A. (1998). Position paper of The American Academy of Periodontology: periodontal disease as a potential risk factor for systemic diseases. *Journal of Periodontology*.

[25] Słotwińska S. M. (2012). Cytokines and periodontitis. Part I: interleukin-1 and interleukin-1 receptor antagonist. *Central European Journal of Immunology*.

[26] Liu Z., Liu Y., Song Y., Zhang X., Wang S., Wang Z. (2014). Systemic oxidative stress biomarkers in chronic periodontitis: a meta-analysis. *Disease Markers*.

[27] Ogawa H., Damrongrungruang T., Hori S. (2014). Effect of periodontal treatment on adipokines in type 2 diabetes. *World Journal of Diabetes*.

[28] Baltacioğlu E., Kehribar M. A., Yuva P. (2014). Total oxidant status and bone resorption biomarkers in serum and gingival crevicular fluid of patients with periodontitis. *Journal of Periodontology*.

[29] Osato J. A., Korkina L. G., Santiago L. A., Afanas'ev I. B. (1995). Effects of bio-normalizer (a food supplementation) on free radical production by human blood neutrophils, erythrocytes, and rat peritoneal macrophages. *Nutrition*.

[30] Mikhalchik E. V., Ivanova A. V., Anurov M. V. (2004). Wound-healing effect of papaya-based preparation in experimental thermal trauma. *Bulletin of Experimental Biology and Medicine*.

[31] Korkina L., Osato J. A., Chivilyeva I., Samochatova E., Cheremisina Z., Afanasev I. (1995). Radioprotective and antioxidant effects of zinc aspartate and bio-normalizer in children with acute myelo- and lympholeukemias. *Nutrition*.

[32] Dickerson R., Deshpande B., Gnyawali U. (2012). Correction of aberrant NADPH oxidase activity in blood-derived mononuclear cells from type II diabetes mellitus patients by a naturally fermented papaya preparation. *Antioxidants and Redox Signaling*.

[33] Dickerson R., Banerjee J., Rauckhorst A. (2015). Does oral supplementation of a fermented papaya preparation correct respiratory burst function of innate immune cells in type 2 diabetes mellitus patients?. *Antioxidants and Redox Signaling*.

[34] Barnett M. L. (1996). Suitability of gingival indices for use in therapeutic trials. Is bleeding a sine qua non?. *Journal of Clinical Periodontology*.

[35] Marks R. G., Magnusson I., Taylor M., Clouser B., Maruniak J., Clark W. B. (1993). Evaluation of reliability and reproducibility of dental indices. *Journal of Clinical Periodontology*.

[36] Aebi H. (1984). Catalase in vitro. *Methods in Enzymology*.

[37] Misra H. P., Fridovich I. (1978). Inhibition of superoxide dismutases by azide. *Archives of Biochemistry and Biophysics*.

[38] Jin C., Flavell R. A. (2013). Innate sensors of pathogen and stress: linking inflammation to obesity. *Journal of Allergy and Clinical Immunology*.

[39] Shrestha D., Choi Y. H., Zhang J., Hazlett L. J., Merchant A. T. (2015). Relationship between serologic markers of periodontal bacteria and metabolic syndrome and its components. *Journal of Periodontology*.

[40] Somanah J., Bourdon E., Bahorun T., Aruoma O. I. (2013). The inhibitory effect of a fermented papaya preparation on growth, hydrophobicity, and acid production of *Streptococcus mutans*, *Streptococcus mitis*, and *Lactobacillus acidophilus*: its implications in oral health improvement of diabetics. *Food Science and Nutrition*.

[41] Osato J. A., Korkina L. G., Santiago L. A., Afanas'ev I. B. (1995). Effects of bio-normalizer (a food supplementation) on free radical production by human blood neutrophils, erythrocytes, and rat peritoneal macrophages. *Nutrition*.

[42] Korkina L., Osato J. A., Chivilyeva I., Samochatova E., Cheremisina Z., Afanas'ev I. (1995). Radioprotective and antioxidant effects of zinc aspartate and bio-normalizer in children with acute myelo- and lympho-leukemias. *Nutrition (Supplements)*.

[43] Osato J. A., Afanas’ev I. B., Cheremisina Z. P. (1995). Bio-normalizer as a modulator of phagocytosis and free radical production by murine inflamed neutrophils and macrophages. *Physics, Chemistry, Biology & Medicine*.

[44] Somanah J., Bourdon E., Rondeau P., Bahorun T., Aruoma O. I. (2014). Relationship between fermented papaya preparation supplementation, erythrocyte integrity and antioxidant status in pre-diabetics. *Food and Chemical Toxicology*.

[45] Chapple I. L. C., Mason G. I., Garner I. (1997). Enhanced chemiluminescent assay for measuring the total antioxidant capacity of serum, saliva and crevicular fluid. *Annals of Clinical Biochemistry*.

[46] Esen Ç., Alkan B. A., Kirnap M., Akgül Ö., Işıkoğlu S., Erel Ö. (2012). The effects of chronic periodontitis and rheumatoid arthritis on serum and gingival crevicular fluid total antioxidant/oxidant status and oxidative stress index. *Journal of Periodontology*.

[47] Dalai C., Ignat-Romanul I., Roşca E. (2013). Correlation between histopathological aspects of periodontitis and biochemical changes of oxidative stress. *Romanian Journal of Morphology and Embryology*.

[48] Lubrano C., Valacchi G., Specchia P. (2015). Integrated haematological profiles of redox *status*, lipid, and inflammatory protein biomarkers in benign obesity and unhealthy obesity with metabolic syndrome. *Oxidative Medicine and Cellular Longevity*.

[49] Goutoudi P., Diza E., Arvanitidou M. (2004). Effect of periodontal therapy on crevicular fluid interleukin-1*β* and interleukin-10 levels in chronic periodontitis. *Journal of Dentistry*.

[50] Kaushik R., Yeltiwar R. K., Pushpanshu K. (2011). Salivary interleukin-1*β* levels in patients with chronic periodontitis before and after periodontal phase i therapy and healthy controls: a case-control study. *Journal of Periodontology*.

[51] Collard E., Roy S. (2010). Improved function of diabetic wound-site macrophages and accelerated wound closure in response to oral supplementation of a fermented papaya preparation. *Antioxidants and Redox Signaling*.

[52] Fibach E., Ginsburg I. (2015). The antioxidant effect of fermented papaya preparation in the oral cavity. *Phytotherapy Research*.

[53] Buommino E., Scognamiglio M., Donnarumma G., Fiorentino A., D’Abrosca B. (2015). Recent advances in natural product-based anti-biofilm approaches to control infections. *Mini-Reviews in Medicinal Chemistry*.

[54] Gaupp R., Ledala N., Somerville G. A. (2012). Staphylococcal response to oxidative stress. *Frontiers in Cellular and Infection Microbiology*.

[55] Karavolos M. H., Horsburgh M., Ingham E., Foster S. J. (2003). Role and regulation of the superoxide dismutases of *Staphylococcus aureus*. *Microbiology*.

[56] Cosgrove K., Coutts G., Jonsson I.-M. (2007). Catalase (KatA) and alkyl hydroperoxide reductase (AhpC) have compensatory roles in peroxide stress resistance and are required for survival, persistence, and nasal colonization in *Staphylococcus aureus*. *Journal of Bacteriology*.

[57] Imlay J. A. (2015). Transcription factors that defend bacteria against reactive oxygen species. *Annual Review of Microbiology*.

[58] Brennan R. E., Kiss K., Baalman R., Samuel J. E. (2015). Cloning, expression, and characterization of a *Coxiella burnetii* Cu/Zn superoxide dismutase. *BMC Microbiology*.

[59] Meuric V., Gracieux P., Tamanai-Shacoori Z., Perez-Chaparro J., Bonnaure-Mallet M. (2008). Expression patterns of genes induced by oxidative stress in *Porphyromonas gingivalis*. *Oral Microbiology and Immunology*.

[60] Dou Y., Aruni W., Luo T., Roy F., Wang C., Fletcher H. M. (2014). Involvement of PG2212 zinc finger protein in the regulation of oxidative stress resistance in *Porphyromonas gingivalis* W83. *Journal of Bacteriology*.

[61] Imlay J. A. (2015). Diagnosing oxidative stress in bacteria: not as easy as you might think. *Current Opinion in Microbiology*.

[62] Scott D. A., Krauss J. (2012). Neutrophils in periodontal inflammation. *Frontiers of Oral Biology*.

